# Dibromido(4,4′-dimethyl-2,2′-bipyridine-κ^2^ *N*,*N*′)(dimethyl sulfoxide-κ*O*)cadmium

**DOI:** 10.1107/S1600536812028553

**Published:** 2012-06-30

**Authors:** Sadif A. Shirvan, Sara Haydari Dezfuli

**Affiliations:** a Department of Chemistry, Islamic Azad University, Omidieh Branch, Omidieh, Iran

## Abstract

In the title compound, [CdBr_2_(C_12_H_12_N_2_)(C_2_H_6_OS)], the Cd^II^ atom is five-coordinated in a distorted trigonal–bipyramidal geometry by two N atoms from one 4,4′-dimethyl-2,2′-bipyridine (DMBP) ligand, one O atom from a dimethyl sulfoxide (DMSO) ligand and two Br atoms. A weak intra­molecular C—H⋯O hydrogen bond occurs between the DMBP and DMSO ligands. π–π stacking between pyridine rings [centroid–centroid distances = 3.682 (3) and 3.598 (3) Å] is observed in the crystal.

## Related literature

For related structures, see: Ahmadi *et al.* (2008 ▶); Alizadeh *et al.* (2010 ▶); Amani *et al.* (2009 ▶); Bellusci *et al.* (2008 ▶); Hojjat Kashani *et al.* (2008 ▶); Kalateh *et al.* (2008 ▶, 2010 ▶); Sakamoto *et al.* (2004 ▶); Shirvan & Haydari Dezfuli (2011 ▶); Sofetis *et al.* (2006 ▶); Willett *et al.* (2001 ▶); Yoshikawa *et al.* (2003 ▶); Yousefi *et al.* (2008 ▶).
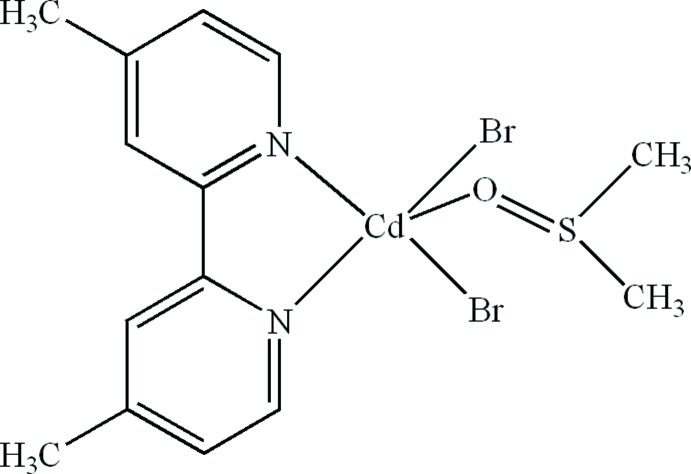


## Experimental

### 

#### Crystal data

[CdBr_2_(C_12_H_12_N_2_)(C_2_H_6_OS)]
*M* *_r_* = 534.58
Monoclinic, 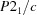

*a* = 8.3940 (6) Å
*b* = 15.2928 (15) Å
*c* = 14.8606 (9) Å
β = 103.377 (5)°
*V* = 1855.9 (3) Å^3^
*Z* = 4
Mo *K*α radiation
μ = 5.59 mm^−1^
*T* = 298 K
0.33 × 0.28 × 0.20 mm

#### Data collection

Bruker APEXII CCD area-detector diffractometer
Absorption correction: multi-scan (*SADABS*; Bruker, 2001 ▶) *T*_min_ = 0.183, *T*_max_ = 0.342
16741 measured reflections
4054 independent reflections
3008 reflections with *I* > 2σ(*I*)
*R*_int_ = 0.099

#### Refinement

*R*[*F* ^2^ > 2σ(*F* ^2^)] = 0.041
*wR*(*F* ^2^) = 0.086
*S* = 1.04
4054 reflections
190 parameters
H-atom parameters constrained
Δρ_max_ = 0.53 e Å^−3^
Δρ_min_ = −0.42 e Å^−3^

### 

Data collection: *APEX2* (Bruker, 2007 ▶); cell refinement: *SAINT* (Bruker, 2007 ▶); data reduction: *SAINT*; program(s) used to solve structure: *SHELXS97* (Sheldrick, 2008 ▶); program(s) used to refine structure: *SHELXL97* (Sheldrick, 2008 ▶); molecular graphics: *SHELXTL* (Sheldrick, 2008 ▶); software used to prepare material for publication: *SHELXTL*.

## Supplementary Material

Crystal structure: contains datablock(s) I, global. DOI: 10.1107/S1600536812028553/xu5571sup1.cif

Structure factors: contains datablock(s) I. DOI: 10.1107/S1600536812028553/xu5571Isup2.hkl

Additional supplementary materials:  crystallographic information; 3D view; checkCIF report

## Figures and Tables

**Table 1:** Selected bond lengths (Å)

Cd1—O1	2.301 (4)
Cd1—N1	2.349 (3)
Cd1—N2	2.340 (3)
Cd1—Br1	2.5857 (6)
Cd1—Br2	2.5784 (6)

**Table 2:** Hydrogen-bond geometry (Å, °)

*D*—H⋯*A*	*D*—H	H⋯*A*	*D*⋯*A*	*D*—H⋯*A*
C12—H12⋯O1	0.93	2.51	3.087 (5)	120

**Fig. 1.**
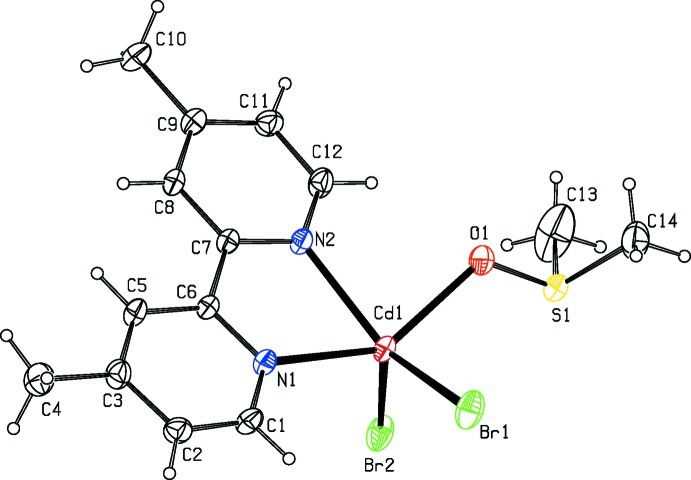
The molecular structure of the title molecule, with the atom-numbering scheme. Displacement ellipsoids are drawn at the 30% probability level.

**Fig. 2.**
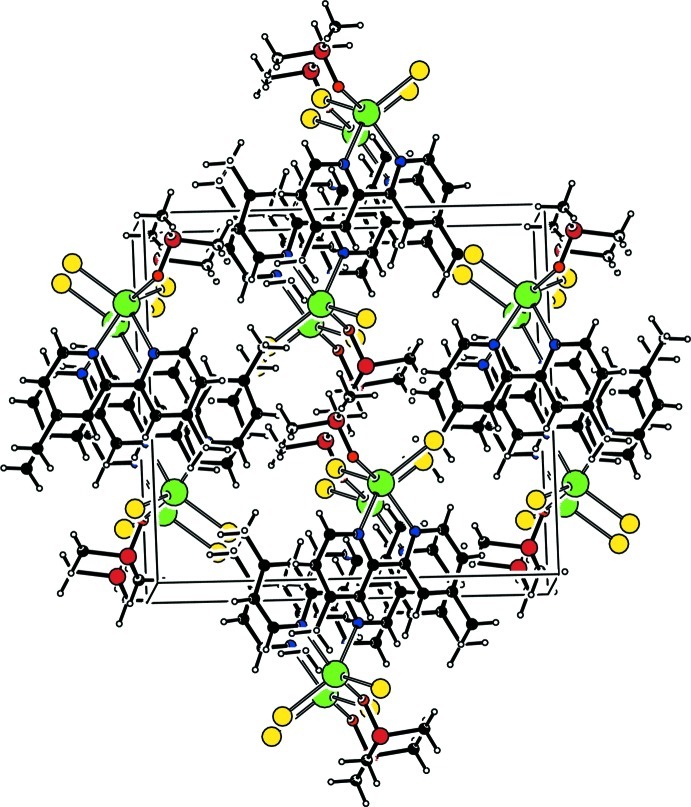
Unit-cell packing diagram for title compound.

## supplementary crystallographic information

### Comment

4,4'-Dimethyl-2,2'-bipyridine (4,4'-dmbipy), is a good bidentate ligand, and numerous complexes with 4,4'-dmbipy have been prepared, such as that of mercury (Kalateh *et al.*, 2008; Yousefi *et al.*, 2008), indium (Ahmadi *et al.*, 2008), iron (Amani *et al.*, 2009), platin (Hojjat Kashani *et al.*, 2008), manganese (Sakamoto *et al.*, 2004), silver (Bellusci *et al.*, 2008), gallium (Sofetis *et al.*, 2006), copper (Willett *et al.*, 2001), iridium (Yoshikawa *et al.*, 2003), cadmium (Kalateh *et al.*, 2010) and zinc (Alizadeh *et al.*, 2010; Shirvan & Haydari Dezfuli, 2011). Here, we report the synthesis and structure of the title compound.

In the title compound, (Fig. 1), the Cd^II^ atom is five-coordinated in a distorted trigonal-bipyramidal configuration by two N atoms from one 4,4'-dimethyl-2,2'-bipyridine, one O atom from one dimethyl sulfoxide and two Br atoms. The Cd—Br and Cd—N bond lengths and angles are collected in Table 1.

In the crystal structure, intermolecular C—H···O hydrogen bonds (Table 2) and π-π contacts (Fig. 2) between the pyridine rings, *Cg*3—*Cg*2^i^ and *Cg*3—*Cg*3^ii^ [symmetry cods: (i) –*X*,-Y,1-*Z* and (ii) 1-*X*,-Y,1-*Z*, where *Cg*2 and *Cg*3 are centroids of the rings (N1/C1—C3/C5—C6) and (N2/C7—C9/C11—C12), respectively] may stabilize the structure, with centroid-centroid distance of 3.682 (3) and 3.598 (3) Å.

### Experimental

For the preparation of the title compound, a solution of 4,4'-dimethyl-2,2'-bipyridine (0.25 g, 1.33 mmol) in methanol (10 ml) was added to a solution of CdBr_2_.4H_2_O, (0.46 g, 1.33 mmol) in methanol (10 ml) at room temperature. The suitable crystals for X-ray diffraction experiment were obtained by methanol diffusion to a colorless solution in DMSO. Suitable crystals were isolated after one week (yield; 0.52 g, 73.1%).

### Refinement

H atoms were positioned geometrically with C—H = 0.93–0.96 Å and constrained to ride on their parent atoms, *U*_iso_(H) = 1.5*U*_eq_(C) for methyl H atoms and 1.2U_eq_(C) for the others.

### Crystal data

**Table:**

[CdBr_2_(C_12_H_12_N_2_)(C_2_H_6_OS)]	*F*(000) = 1032
*M**_r_* = 534.58	*D*_x_ = 1.913 Mg m^−^^3^
Monoclinic, *P*2_1_/*c*	Mo *K*α radiation, λ = 0.71073 Å
Hall symbol: -P 2ybc	Cell parameters from 10741 reflections
*a* = 8.3940 (6) Å	θ = 1.9–27.0°
*b* = 15.2928 (15) Å	µ = 5.59 mm^−^^1^
*c* = 14.8606 (9) Å	*T* = 298 K
β = 103.377 (5)°	Block, colorless
*V* = 1855.9 (3) Å^3^	0.33 × 0.28 × 0.20 mm
*Z* = 4	

### Data collection

**Table:**

Bruker APEXII CCD area-detector diffractometer	4054 independent reflections
Radiation source: fine-focus sealed tube	3008 reflections with *I* > 2σ(*I*)
Graphite monochromator	*R*_int_ = 0.099
ω scans	θ_max_ = 27.0°, θ_min_ = 1.9°
Absorption correction: multi-scan (*SADABS*; Bruker, 2001)	*h* = −9→10
*T*_min_ = 0.183, *T*_max_ = 0.342	*k* = −19→19
16741 measured reflections	*l* = −18→18

### Refinement

**Table:**

Refinement on *F*^2^	Primary atom site location: structure-invariant direct methods
Least-squares matrix: full	Secondary atom site location: difference Fourier map
*R*[*F*^2^ > 2σ(*F*^2^)] = 0.041	Hydrogen site location: inferred from neighbouring sites
*wR*(*F*^2^) = 0.086	H-atom parameters constrained
*S* = 1.04	*w* = 1/[σ^2^(*F*_o_^2^) + (0.0279*P*)^2^ + 0.8746*P*] where *P* = (*F*_o_^2^ + 2*F*_c_^2^)/3
4054 reflections	(Δ/σ)_max_ = 0.010
190 parameters	Δρ_max_ = 0.53 e Å^−^^3^
0 restraints	Δρ_min_ = −0.42 e Å^−^^3^

### Special details

**Table:**

Geometry. All e.s.d.'s (except the e.s.d. in the dihedral angle between two l.s. planes) are estimated using the full covariance matrix. The cell e.s.d.'s are taken into account individually in the estimation of e.s.d.'s in distances, angles and torsion angles; correlations between e.s.d.'s in cell parameters are only used when they are defined by crystal symmetry. An approximate (isotropic) treatment of cell e.s.d.'s is used for estimating e.s.d.'s involving l.s. planes.
Refinement. Refinement of *F*^2^ against ALL reflections. The weighted *R*-factor *wR* and goodness of fit *S* are based on *F*^2^, conventional *R*-factors *R* are based on *F*, with *F* set to zero for negative *F*^2^. The threshold expression of *F*^2^ > σ(*F*^2^) is used only for calculating *R*-factors(gt) *etc*. and is not relevant to the choice of reflections for refinement. *R*-factors based on *F*^2^ are statistically about twice as large as those based on *F*, and *R*- factors based on ALL data will be even larger.

### Fractional atomic coordinates and isotropic or equivalent isotropic displacement parameters (Å^2^)

**Table:**

	*x*	*y*	*z*	*U*_iso_*/*U*_eq_	
C1	0.0502 (6)	0.2148 (3)	0.6246 (3)	0.0501 (11)	
H1	0.0521	0.2350	0.6838	0.060*	
C2	−0.0370 (7)	0.2624 (3)	0.5505 (3)	0.0526 (12)	
H2	−0.0933	0.3127	0.5599	0.063*	
C3	−0.0391 (7)	0.2337 (3)	0.4611 (3)	0.0528 (12)	
C4	−0.1294 (8)	0.2827 (4)	0.3769 (4)	0.0767 (18)	
H4A	−0.0862	0.3409	0.3779	0.092*	
H4B	−0.2437	0.2854	0.3769	0.092*	
H4C	−0.1160	0.2529	0.3223	0.092*	
C5	0.0474 (6)	0.1592 (3)	0.4529 (3)	0.0473 (11)	
H5	0.0502	0.1387	0.3944	0.057*	
C6	0.1305 (5)	0.1138 (2)	0.5302 (2)	0.0373 (9)	
C7	0.2243 (5)	0.0322 (2)	0.5237 (3)	0.0365 (9)	
C8	0.2297 (6)	−0.0049 (3)	0.4393 (3)	0.0426 (10)	
H8	0.1732	0.0214	0.3848	0.051*	
C9	0.3177 (6)	−0.0803 (3)	0.4350 (3)	0.0476 (11)	
C10	0.3268 (7)	−0.1202 (3)	0.3438 (3)	0.0634 (14)	
H10A	0.2846	−0.1787	0.3402	0.076*	
H10B	0.4387	−0.1213	0.3387	0.076*	
H10C	0.2629	−0.0858	0.2943	0.076*	
C11	0.3983 (7)	−0.1162 (3)	0.5174 (3)	0.0535 (12)	
H11	0.4593	−0.1670	0.5178	0.064*	
C12	0.3894 (7)	−0.0776 (3)	0.5995 (3)	0.0539 (12)	
H12	0.4446	−0.1034	0.6545	0.065*	
C13	0.5760 (15)	−0.1667 (4)	0.8786 (5)	0.144 (4)	
H13A	0.6672	−0.1729	0.8502	0.173*	
H13B	0.4795	−0.1906	0.8385	0.173*	
H13C	0.5987	−0.1973	0.9365	0.173*	
C14	0.7460 (8)	−0.0254 (5)	0.9509 (4)	0.094 (2)	
H14A	0.7545	0.0372	0.9532	0.112*	
H14B	0.8195	−0.0483	0.9159	0.112*	
H14C	0.7744	−0.0485	1.0126	0.112*	
N1	0.1310 (5)	0.1425 (2)	0.6162 (2)	0.0418 (8)	
N2	0.3045 (5)	−0.0041 (2)	0.6035 (2)	0.0424 (8)	
Br1	0.35504 (9)	0.19185 (4)	0.84879 (4)	0.0779 (2)	
Br2	0.07055 (8)	−0.04712 (4)	0.79461 (3)	0.06865 (18)	
O1	0.5107 (5)	−0.0161 (3)	0.8016 (2)	0.0663 (10)	
S1	0.54455 (17)	−0.05549 (9)	0.89770 (7)	0.0547 (3)	
Cd1	0.27102 (4)	0.05814 (2)	0.741841 (19)	0.04315 (10)	

### Atomic displacement parameters (Å^2^)

**Table:**

	*U*^11^	*U*^22^	*U*^33^	*U*^12^	*U*^13^	*U*^23^
C1	0.065 (3)	0.048 (2)	0.040 (2)	0.002 (2)	0.017 (2)	−0.0086 (18)
C2	0.060 (3)	0.046 (2)	0.053 (3)	0.011 (2)	0.016 (2)	−0.002 (2)
C3	0.062 (3)	0.051 (3)	0.043 (2)	0.005 (2)	0.007 (2)	0.0069 (19)
C4	0.094 (5)	0.076 (4)	0.058 (3)	0.032 (3)	0.014 (3)	0.017 (3)
C5	0.060 (3)	0.051 (2)	0.030 (2)	0.000 (2)	0.0084 (19)	0.0015 (17)
C6	0.043 (2)	0.040 (2)	0.0296 (19)	−0.0028 (18)	0.0094 (17)	−0.0016 (15)
C7	0.042 (2)	0.0367 (19)	0.0315 (18)	−0.0094 (17)	0.0090 (17)	−0.0005 (14)
C8	0.049 (3)	0.048 (2)	0.0295 (19)	−0.003 (2)	0.0061 (18)	0.0004 (16)
C9	0.057 (3)	0.047 (2)	0.039 (2)	−0.004 (2)	0.012 (2)	−0.0054 (17)
C10	0.083 (4)	0.067 (3)	0.045 (3)	0.001 (3)	0.024 (3)	−0.011 (2)
C11	0.062 (3)	0.050 (3)	0.051 (3)	0.012 (2)	0.017 (2)	−0.003 (2)
C12	0.069 (3)	0.050 (3)	0.039 (2)	0.009 (2)	0.006 (2)	0.0047 (18)
C13	0.262 (13)	0.059 (4)	0.091 (5)	0.014 (6)	0.003 (7)	0.010 (3)
C14	0.075 (5)	0.144 (6)	0.055 (3)	−0.019 (4)	0.001 (3)	0.000 (4)
N1	0.050 (2)	0.0409 (18)	0.0332 (17)	−0.0013 (17)	0.0076 (16)	−0.0039 (14)
N2	0.050 (2)	0.0457 (19)	0.0309 (16)	0.0009 (17)	0.0077 (15)	0.0003 (14)
Br1	0.1001 (5)	0.0708 (3)	0.0542 (3)	−0.0152 (3)	0.0004 (3)	−0.0206 (2)
Br2	0.0777 (4)	0.0856 (4)	0.0417 (3)	−0.0271 (3)	0.0118 (2)	0.0113 (2)
O1	0.066 (2)	0.091 (2)	0.0404 (17)	0.022 (2)	0.0083 (16)	0.0151 (17)
S1	0.0557 (7)	0.0726 (7)	0.0358 (5)	0.0043 (7)	0.0102 (5)	0.0008 (5)
Cd1	0.0511 (2)	0.04968 (17)	0.02831 (14)	−0.00105 (16)	0.00838 (12)	0.00028 (12)

### Geometric parameters (Å, º)

**Table:**

C1—N1	1.318 (6)	C10—H10B	0.9600
C1—C2	1.380 (7)	C10—H10C	0.9600
C1—H1	0.9300	C11—C12	1.372 (6)
C2—C3	1.394 (6)	C11—H11	0.9300
C2—H2	0.9300	C12—N2	1.339 (6)
C3—C5	1.373 (7)	C12—H12	0.9300
C3—C4	1.504 (7)	C13—S1	1.754 (7)
C4—H4A	0.9600	C13—H13A	0.9600
C4—H4B	0.9600	C13—H13B	0.9600
C4—H4C	0.9600	C13—H13C	0.9600
C5—C6	1.384 (6)	C14—S1	1.755 (6)
C5—H5	0.9300	C14—H14A	0.9600
C6—N1	1.351 (5)	C14—H14B	0.9600
C6—C7	1.489 (6)	C14—H14C	0.9600
C7—N2	1.342 (5)	Cd1—O1	2.301 (4)
C7—C8	1.387 (5)	Cd1—N1	2.349 (3)
C8—C9	1.378 (6)	Cd1—N2	2.340 (3)
C8—H8	0.9300	Cd1—Br1	2.5857 (6)
C9—C11	1.370 (6)	Cd1—Br2	2.5784 (6)
C9—C10	1.505 (6)	O1—S1	1.515 (3)
C10—H10A	0.9600		
			
N1—C1—C2	123.8 (4)	C9—C11—H11	119.8
N1—C1—H1	118.1	C12—C11—H11	119.8
C2—C1—H1	118.1	N2—C12—C11	122.7 (4)
C1—C2—C3	118.8 (4)	N2—C12—H12	118.7
C1—C2—H2	120.6	C11—C12—H12	118.7
C3—C2—H2	120.6	S1—C13—H13A	109.5
C5—C3—C2	117.1 (4)	S1—C13—H13B	109.5
C5—C3—C4	121.0 (4)	H13A—C13—H13B	109.5
C2—C3—C4	121.9 (5)	S1—C13—H13C	109.5
C3—C4—H4A	109.5	H13A—C13—H13C	109.5
C3—C4—H4B	109.5	H13B—C13—H13C	109.5
H4A—C4—H4B	109.5	S1—C14—H14A	109.5
C3—C4—H4C	109.5	S1—C14—H14B	109.5
H4A—C4—H4C	109.5	H14A—C14—H14B	109.5
H4B—C4—H4C	109.5	S1—C14—H14C	109.5
C3—C5—C6	121.2 (4)	H14A—C14—H14C	109.5
C3—C5—H5	119.4	H14B—C14—H14C	109.5
C6—C5—H5	119.4	C1—N1—C6	118.3 (4)
N1—C6—C5	120.8 (4)	C1—N1—Cd1	124.0 (3)
N1—C6—C7	116.6 (3)	C6—N1—Cd1	117.7 (3)
C5—C6—C7	122.6 (3)	C12—N2—C7	118.1 (3)
N2—C7—C8	121.0 (4)	C12—N2—Cd1	123.6 (3)
N2—C7—C6	117.0 (3)	C7—N2—Cd1	118.1 (3)
C8—C7—C6	122.0 (4)	S1—O1—Cd1	121.1 (2)
C9—C8—C7	120.9 (4)	O1—S1—C13	103.6 (3)
C9—C8—H8	119.5	O1—S1—C14	105.7 (3)
C7—C8—H8	119.5	C13—S1—C14	99.3 (5)
C11—C9—C8	116.9 (4)	O1—Cd1—N2	82.34 (12)
C11—C9—C10	121.7 (4)	O1—Cd1—N1	144.06 (13)
C8—C9—C10	121.3 (4)	N2—Cd1—N1	70.41 (12)
C9—C10—H10A	109.5	O1—Cd1—Br2	98.54 (10)
C9—C10—H10B	109.5	N2—Cd1—Br2	103.45 (9)
H10A—C10—H10B	109.5	N1—Cd1—Br2	110.05 (9)
C9—C10—H10C	109.5	O1—Cd1—Br1	93.61 (10)
H10A—C10—H10C	109.5	N2—Cd1—Br1	142.22 (9)
H10B—C10—H10C	109.5	N1—Cd1—Br1	93.99 (8)
C9—C11—C12	120.3 (4)	Br2—Cd1—Br1	114.28 (2)
			
N1—C1—C2—C3	−0.9 (8)	C8—C7—N2—C12	−0.3 (6)
C1—C2—C3—C5	−0.2 (7)	C6—C7—N2—C12	179.9 (4)
C1—C2—C3—C4	−179.2 (5)	C8—C7—N2—Cd1	−175.5 (3)
C2—C3—C5—C6	1.1 (7)	C6—C7—N2—Cd1	4.6 (5)
C4—C3—C5—C6	−179.9 (5)	Cd1—O1—S1—C13	122.3 (5)
C3—C5—C6—N1	−1.0 (7)	Cd1—O1—S1—C14	−133.7 (3)
C3—C5—C6—C7	179.4 (4)	S1—O1—Cd1—N2	−145.6 (3)
N1—C6—C7—N2	−1.8 (6)	S1—O1—Cd1—N1	174.01 (19)
C5—C6—C7—N2	177.7 (4)	S1—O1—Cd1—Br2	−43.1 (3)
N1—C6—C7—C8	178.4 (4)	S1—O1—Cd1—Br1	72.1 (3)
C5—C6—C7—C8	−2.1 (6)	C12—N2—Cd1—O1	24.8 (4)
N2—C7—C8—C9	0.0 (7)	C7—N2—Cd1—O1	−160.3 (3)
C6—C7—C8—C9	179.8 (4)	C12—N2—Cd1—N1	−179.0 (4)
C7—C8—C9—C11	0.1 (7)	C7—N2—Cd1—N1	−4.1 (3)
C7—C8—C9—C10	−178.9 (5)	C12—N2—Cd1—Br2	−72.2 (4)
C8—C9—C11—C12	0.1 (8)	C7—N2—Cd1—Br2	102.7 (3)
C10—C9—C11—C12	179.1 (5)	C12—N2—Cd1—Br1	110.8 (4)
C9—C11—C12—N2	−0.4 (8)	C7—N2—Cd1—Br1	−74.3 (4)
C2—C1—N1—C6	1.0 (7)	C1—N1—Cd1—O1	−135.5 (4)
C2—C1—N1—Cd1	−177.5 (4)	C6—N1—Cd1—O1	46.0 (4)
C5—C6—N1—C1	0.0 (6)	C1—N1—Cd1—N2	−178.5 (4)
C7—C6—N1—C1	179.5 (4)	C6—N1—Cd1—N2	3.1 (3)
C5—C6—N1—Cd1	178.5 (3)	C1—N1—Cd1—Br2	83.9 (4)
C7—C6—N1—Cd1	−1.9 (5)	C6—N1—Cd1—Br2	−94.6 (3)
C11—C12—N2—C7	0.5 (7)	C1—N1—Cd1—Br1	−33.8 (4)
C11—C12—N2—Cd1	175.5 (4)	C6—N1—Cd1—Br1	147.8 (3)

### Hydrogen-bond geometry (Å, º)

**Table:**

*D*—H···*A*	*D*—H	H···*A*	*D*···*A*	*D*—H···*A*
C12—H12···O1	0.93	2.51	3.087 (5)	120
